# Editorial: Immunotherapy as an Evolving Approach for the Treatment of Breast Cancer

**DOI:** 10.3389/fonc.2021.752689

**Published:** 2021-11-24

**Authors:** Mai F. Tolba, Cesar A. Santa-Maria, Adriana Albini, Emile R. Chimusa, Basel K. Al-Ramadi, Sara M. Tolaney

**Affiliations:** ^1^ Department of Pharmacology and Toxicology, Faculty of Pharmacy, Ain Shams University, Cairo, Egypt; ^2^ School of Life and Medical Sciences, University of Hertfordshire Hosted by Global Academic Foundation (GAF), New Administrative Capital, Cairo, Egypt; ^3^ Sidney Kimmel Comprehensive Cancer Center, Johns Hopkins Medicine, Baltimore, MD, United States; ^4^ Laboratory of Vascular Biology and Angiogenesis, Istituto di Ricovero e Cura a Carattere Scientifico (IRCCS) MultiMedica, Milan, Italy; ^5^ Division of Human Genetics, Department of Pathology, Institute of Infectious Disease and Molecular Medicine, Faculty of Health Science, University of Cape Town, Cape Town, South Africa; ^6^ Department of Medical Microbiology and Immunology, College of Medicine and Health Sciences, United Arab Emirates University, Al Ain, United Arab Emirates; ^7^ Zayed Center for Health Sciences, United Arab Emirates University, Al Ain, United Arab Emirates; ^8^ Department of Medical Oncology, Dana-Farber Cancer Institute, Boston, MA, United States; ^9^ Department of Medicine, Harvard Medical School, Boston, MA, United States

**Keywords:** immunotherapy, breast cancer, immune checkpoint inhibitors, combination therapy, biomarker

Novel therapies have improved outcomes of breast cancer (BC) patients, but still many progress to the metastatic disease, which remains very difficult to cure. Hormonal and targeted therapies including monoclonal antibodies against HER2 have become routine treatment in BC. In recent years oncology has made great advances by tackling the immune system as a new pillar for cancer therapy. Initial work exploring immunotherapy focused on triple-negative breast cancer (TNBC) since it was known to have higher rates of PD-L1 expression, higher prevalence of tumor-infiltrating lymphocytes (TILs), and higher mutational burden (1). Clinical trials for antibodies targeting PD-1/PD-L1 in metastatic TNBC have demonstrated promising therapeutic outcomes (1, 2). Despite a very modest response rate to checkpoint inhibition as monotherapy in TNBC, patients who achieved response were found to have prolonged overall survival (1). Therefore, the main challenge is to develop strategies to boost the tumor response to immunotherapy in order to increase the percentage of patients benefiting from therapy. The beginning of 2019 witnessed the first FDA accelerated approval of immunotherapy for the treatment of patients with metastatic PD-L1+ TNBC (3, 4). The first approved combination comprises atezolizumab (anti-PD-L1 monoclonal antibody) together with nab-paclitaxel chemotherapy. This combination represented the first step of introducing immunotherapy to the standard treatment protocol of breast cancer and revolutionized the landscape of treatment for metastatic TNBC. We have since seen approval for pembrolizumab in combination with chemotherapy for first line PD-L1+ metastatic TNBC based on the KEYNOTE-355 study (5), and even more recently we now have approval for pembrolizumab with chemotherapy for the treatment of early stage TNBC, based on results from the KEYNOTE-522 trial (6). While there is a benefit in adding checkpoint inhibitors to chemotherapy in TNBC, not all patients respond to immunotherapy. This has underscored the need for novel strategies to expand the benefits of checkpoint inhibitors for broader populations of patients including patients with advanced hormone receptor-positive (HR+) BC as well as HER-2 positive tumors that are refractory to the standard therapy (2). Early data generated from immunotherapy studies with those other BC subtypes showed clues of improved therapeutic outcomes potentially within certain subsets of patients (2). There are therefore several registration studies for early-stage HR+ disease along with early and advanced HER2+ disease. Moreover, the development of biomarker predictors of benefit and resistance to immunotherapy remains one of the top research priorities for optimizing the application of cancer immunotherapy in the different patient cohorts.

The articles published under this Research Topic fall into two sections. Section I includes articles presenting basic research outcomes or literature reviews highlighting molecular targets and pathways to be tackled to enhance the tumor response to immune checkpoint inhibitors, while section II comprises articles providing rationale for newly established BC immunotherapy clinical trials and/or preliminary outcomes.

## Section I Studies

The articles under this section provide an overview of novel approaches to be adopted in order to enhance the tumor response to immunotherapy. In contrast to normal cells, cancer cells display rapidly adaptive responses to the conditions of oxygen and nutrient insufficiency in a cell survival tactic known as “Metabolic Reprogramming” (7, 8). These changes of tumor cellular bioenergetics include the switch to aerobic glycolysis, a phenomenon known as Warburg effect, are essential for tumor development, invasion, metastasis and resistance to therapies (8). TNBC is known to be a highly glycolytic tumor, providing fuel for growth-promoting biosynthetic pathways and exhibits elevated glucose uptake and a glycolytic gene-expression signature (9, 10). This cancer subtype generates an immunosuppressive tumor microenvironment which is hostile for T-cells and contributes to TNBC immune evasion (11). Thus metabolic reprograming is an attractive approach to reshape the tumor immune environment and bypass immune evasion (Naik and Decock). Moreover, several studies offered an extensive overview for novel molecular targets beyond PD-1/PD-L1 such as ITM2A, VEGFR, STING, TLRs and others (Zhang et al.; Tabana et al.). Other studies discussed the significance of cutting the crosstalk between the tumor cells and other components within the tumor microenvironment (TME) including immune cells, extracellular matrix components and others (Salemme et al.; Deligne and Midwood). Finally, two comprehensive literature reviews underscored the promise of cellular immunotherapies as well as a series of immunotherapy combinations under development for TNBC (Fuentes-Antrás et al.; Thomas et al.).

Naik and Decock discussed how tumor metabolism shapes the local immune environment, with particular emphasis on the aerobic glycolysis-coupled lactate metabolism in TNBC. In addition to the well-established role of metabolic reprograming in accelerating tumor cell proliferation, invasion, metastasis and angiogenesis, the review highlighted the immunosuppressive effects of a lactate-rich microenvironment through modulation of tumor-infiltrating T-cells, natural killer (NK) cells, dendritic cells, Tregs and myeloid-derived suppressor cells as well as tumor-associated macrophages. These data support the rationale for targeting intra-tumoral metabolic landscape to augment the anti-tumor response to immunotherapy and improve the outcomes in highly glycolytic tumors such as TNBC.

Zhang et al. contributed an original research article in which they investigated the tumor suppressor role of the integral membrane protein 2A (ITM2A) in BC and how it is correlated to PD-L1 expression. This study showed that the differentially expressed genes (DEGs) screened based on RNA-sequencing data of MCF-7 cells overexpressing ITM2A were associated with immune response. ITM2A was shown to induce PD-L1 expression in BC cells and boost TILs numbers in the tumor microenvironment. The authors concluded that the overexpression of ITM2A reduced the aggressiveness of BC cells and had a favorable effect on outcomes in BC patients.

Tabana et al. reviewed novel immunological targets beyond PD-1/PD-L1 axis that can be exploited to tune up the tumor immune microenvironment and enhance the outcomes of immunotherapies. Those included engaging stimulator of interferon (IFN) genes (STING), toll like receptors (TLRs), vascular endothelial growth factor receptor (VEGFR) signaling, cytokines along with cyclooxygenase-II (COXII)/prostaglandin E2 (PEG2) axis. Tackling CSF-1/CSF-1R axis as well as adenosine signaling also showed promising outcomes. The modulation of tryptophane and arginine catabolism using inhibitors for indoleamine-2,3-dioxygenase (IDO1) and tryptophan-2,3-dioxygenase (TDO), and arginase 1 was also covered.

Salemme et al. depicted the crosstalk between the tumor cells and the immune TME in BC. In particular the authors presented an updated view of the pro- and anti-tumor activities of the main immune cell populations present in breast TME, with emphasis on the role of cytokine-signaling, cell–cell contact- and microvesicle-based mechanisms. Additionally, this review highlighted the current clinical trials assessing the efficacy of investigational strategies proposed to revert immunosuppression such as chimeric antigen receptor (CAR)-T and CAR-NK cells, cancer vaccination, immunogenic cell death-inducing chemotherapy, DNA methyl transferase and histone deacetylase inhibitors, cytokines or their inhibitors and other immunotherapies in BC.

Deligne and Midwood discussed the controversial role of macrophages and extracellular matrix in BC. Extracellular matrix (ECM) molecules such as tenascin-C, fibronectin and collagen are commonly upregulated within the tumor stroma. Such molecules were reported to exert a complex influence over the behavior of tumor-associated macrophages (TAM). They can either restrict or enhance TAMs intra-tumoral infiltration and drive their polarization towards or away from a pro-tumoral phenotype. On the other hand, TAMs can modulate the production of matrix molecules within the tumor to augment tumor growth and metastasis. The authors suggested that targeting specific immunomodulatory domains of the ECM to reinstate an efficient anti-tumor immune response, and effectively control tumor growth and spread, is emerging as a promising approach offering a new angle in the management of BC.

Fuentes-Antrás et al. outlined a clinically oriented overview of preclinical and clinical data regarding the use of cellular immunotherapies in BC. Cellular therapies aim to harness the immune system as a tool against antigenic heterogeneity and the broad repertoire of immune escape mechanisms occurring in advanced BC. This approach encompasses multiple strategies including the adoptive transfer of TILs, dendritic cells, NK cells, and engineered immune components such as CAR constructs and engineered T cell receptors.

Thomas et al. demonstrated multiple promising future combinations of immune-checkpoint inhibitors in TNBC. The article focused on assessing combinatorial approaches utilizing immune checkpoint inhibitors to enhance both innate and adaptive immune responses, or to establish a more immune favorable environment for cancer vaccines. This article also highlighted the limitations for predictive biomarkers of immunotherapy response. The authors concluded that combination of predictive biomarkers such as PD-L1 expression, intra-tumoral TILs, and stromal TILs density together with tumor mutational burden (TMB), TCR diversity and immune gene signatures will more likely yield improved performance versus each of these biomarkers alone which warrants further investigation.

## Section II Studies

This section includes articles providing rationale for newly established breast cancer immunotherapy clinical trials and/or preliminary outcomes. Among those is the SOLTI-1805 TOT-HER3 trial that focuses on patients with HR+/HER2- BC as well as the PELICAN-IPC trial which focused on HER2- inflammatory BC. This is in addition to the BrEAsT study which investigated immunotherapy/HDACI combination in both TNBC and HER2+ metastatic BC.

Pascual et al. presented the ongoing early phase 1 trial “SOLTI-1805 TOT-HER3”. In this window-of-opportunity study the human epidermal growth factor receptor 3 (HER3)-directed antibody-drug conjugate (ADC) patritumab deruxtecan is given to patients with early-stage hormone receptor-positive (HR+)/human epidermal growth factor receptor 2-negative (HER2-) breast cancer. The primary endpoint is the CelTIL score, a novel tumor microenvironment (TME) biomarker based on the percentage of tumor cellularity and stromal TILs.

Bertucci et al. contributed the rationale and design of the PELICAN-IPC 2015-016/Oncodistinct-003 study (NCT03515798) which is an open-label, randomized, non-comparative, phase II study. PELICAN-IPC is assessing the efficacy, and safety of pembrolizumab in combination with chemotherapy in the neoadjuvant setting in HER2-negative inflammatory breast cancer (IBC). This type of breast cancer is extremely aggressive and is known for very low long-term survival. The mainstay for IBC management was through deploying neoadjuvant chemotherapy protocol. Adding panitumumab (anti-EGFR mAb) to the routine chemotherapy backbone has shown promising outcomes in the HR-/HER2- IBC (12). The PELICAN-IPC trial is the first one to investigate the efficacy of immune checkpoint inhibitors specifically in patients with IBC which is a hard-to-treat form of BC. It is noteworthy that the PELICAN-IPC 2015-016 trial is ongoing, and the estimated study completion date is by 2022.

Gatti-Mays et al. presented the supporting preclinical data and the design of the BrEAsT phase 1b clinical trial (NCT04296942). The study is enrolling patients with advanced/metastatic TNBC or HR-/HER2+ to receive a tetratherapy combination: BN-Brachyury (a poxvirus vaccine encoding a tumor-associated antigen), bintrafusp alfa (a bifunctional protein composed of the extracellular domain of the TGF receptor fused to a human IgG1 anti-PD-L1), entinostat (a histone deacetylase inhibitor), and the HER2-directed ADC ado-trastuzumab emtansine. The study is designed to assess the safety and efficacy of the combination.

Kim et al. contributed a case series of 5 patients with metaplastic BC treated with anti-PD-1-based therapy at a single center. Metaplastic breast cancer (MBC) is known to be a rare and chemo-refractory subtype of BC with poor prognosis and limited treatment options. It is noteworthy that 3 out of the 5 cases demonstrated a response to therapy, albeit limited in duration. One of the responding cases exhibited low-level hormone receptor expression and pleomorphic lobular features, whereas the other cases were TNBC. Responses were observed in tumors with intermediate PD-L1 expression (CPS 1-10). The extensive characterization of MBC was not feasible due to the small sample size in this series. However, in this series the authors also demonstrated a method of interrogating for unique immunologic and/or genomic features of individual tumor cases, relative to a parent cohort.

Schreiber et al. provided a retrospective analysis for the clinical outcomes for patients with metastatic BC treated with immunotherapy agents in Phase I clinical trials. A total of 43 patients with different BC subtypes were identified to be treated with an immunotherapy agent as single agent (72.1%) or combined with chemotherapy (27.9%). All patients had received an average of 2 prior lines of chemotherapy in the metastatic setting. The analysis showed that patients who had a progression-free survival (PFS) of >6 months were more likely to have been treated with a combination of immunotherapy plus chemotherapy compared to patients with a PFS < 6 months (77.8% v. 14.7%), demonstrating the added benefit of using chemotherapy in combination with immunotherapy in metastatic BC irrespective of BC subtype.

In summary, immunotherapy continues to represent an attractive option for patients with TNBC, with emerging strategies being explored in the different subtypes of BC. The emerging data elucidated additional angles for the complex interplay between the different components of the TME along with the ECM and how that contributes to the tumor immune escape. This largely contributes to developing promising strategies that simultaneously target multiple key pathways in order to enhance the therapeutic outcomes for immunotherapies. Yet, further research is still necessary to determine the mechanisms of resistance, identify predictive biomarkers, and to develop optimal combination regimens. These efforts are ongoing in order to provide the most effective, least toxic regimens to the patients that are most likely to benefit.

## Author Contributions

MFT drafted the editorial. SMT, AA, CS, and BA-R contributed comments on the manuscripts they edited. All editors revised and approved the final copy of the editorial before submission.

## Conflict of Interest

SMT receives institutional research funding from AstraZeneca, Lilly, Merck, Nektar, Novartis, Pfizer, Genentech/Roche, Immunomedics/Gilead, Exelixis, Bristol-Myers Squibb, Eisai, Nanostring, Cyclacel, Odonate, and Seattle Genetics; has served as an advisor/consultant to AstraZeneca, Eli Lilly, Merck, Nektar, Novartis, Pfizer, Genentech/Roche, Immunomedics/ Gilead, Bristol-Myers Squibb, Eisai, Nanostring, Puma, Sanofi, Puma, Silverback Therapeutics, G1 Therapeutics, Athenex, OncoPep, Kyowa Kirin Pharmaceuticals, Daiichi-Sankyo, Ellipsis, Infinity, 4D Pharma, and Samsung Bioepsis Inc., Chugai Pharmaceuticals, BeyondSpring Pharmaceuticals, OncXerna, OncoSec Medical Incorporated, Certara, Mersana Therapeutics, CytomX, Seattle Genetics.

The remaining authors declare that the research was conducted in the absence of any commercial or financial relationships that could be construed as a potential conflict of interest.

## Publisher’s Note

All claims expressed in this article are solely those of the authors and do not necessarily represent those of their affiliated organizations, or those of the publisher, the editors and the reviewers. Any product that may be evaluated in this article, or claim that may be made by its manufacturer, is not guaranteed or endorsed by the publisher.
